# Solvent Sorption-Induced Actuation of Composites Based on a Polymer of Intrinsic Microporosity

**DOI:** 10.1021/acsapm.0c01215

**Published:** 2021-01-06

**Authors:** Katarzyna Polak-Kraśna, Mi Tian, Sébastien Rochat, Nicholas Gathercole, Chenggang Yuan, Zhe Hao, Min Pan, Andrew D. Burrows, Timothy J. Mays, Chris R. Bowen

**Affiliations:** † Department of Mechanical Engineering, University of Bath, Claverton Down, Bath BA2 7AY, United Kingdom; ‡ Department of Chemical Engineering, University of Bath, Claverton Down, Bath BA2 7AY, United Kingdom; § Department of Chemistry, University of Bath, Claverton Down, Bath BA2 7AY, United Kingdom; ∥ College of Engineering, Mathematics and Physical Sciences, University of Exeter, Exeter EX4 4QF, United Kingdom; ⊥ School of Chemistry and Bristol Composites Institute (ACCIS), University of Bristol, Bristol BS8 1TH, United Kingdom

**Keywords:** actuator, polymer composite, polymer of intrinsic microporosity, drug delivery, micro-origami capsule

## Abstract

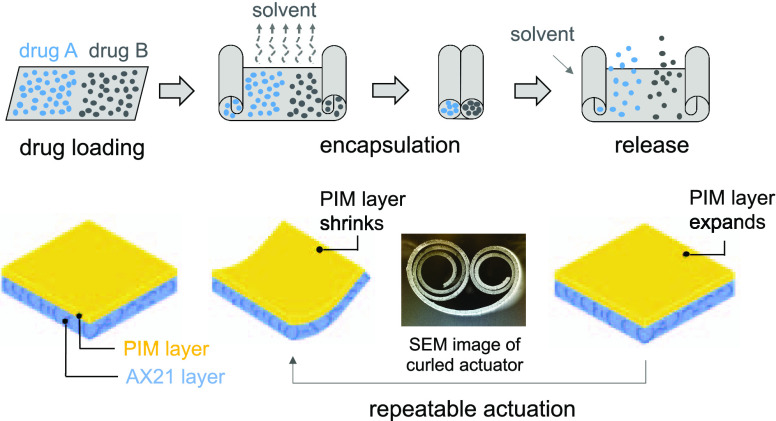

Materials that are capable of actuation in response to a variety of external stimuli are of significant interest for applications in sensors, soft robotics, and biomedical devices. Here, we present a class of actuators using composites based on a polymer of intrinsic microporosity (PIM). By adding an activated carbon (AX21) filler to a PIM, the composite exhibits repeatable actuation upon solvent evaporation and wetting and it is possible to achieve highly controlled three-dimensional actuation. Curled composite actuators are shown to open upon exposure to a solvent and close as a result of solvent evaporation. The degree of curling and actuation is controlled by adjusting the amount of filler and evaporation rate of the solvent casting process, while the actuation speed is controlled by adjusting the type of solvent. The range of forces and actuation speed produced by the composite is demonstrated using acetone, ethanol, and dimethyl sulfoxide as the solvent. The maximum contractile stress produced upon solvent desorption in the pure PIM polymer reached 12 MPa, with an ultimate force over 20 000 times the weight of a sample. This form of the composite actuator is insensitive to humidity and water, which makes it applicable in an aqueous environment, and can survive a wide range of temperatures. These characteristics make it a promising actuator for the diverse range of operating conditions in robotic and medical applications. The mechanism of actuation is discussed, which is based on the asymmetric distribution of the carbon filler particles that leads to a bilayer structure and the individual layers expand and contract differently in response to solvent wetting and evaporation, respectively. Finally, we demonstrate the application of the actuator as a potential drug delivery vehicle, with capacity for encapsulating two kinds of drugs and reduced drug leakage in comparison to existing technologies.

## 1 Introduction

Polymers that are able to react to external stimuli by changing their properties have recently attracted interest due to their applications in biomedicine, mechanical actuation, sensing, soft robotics, and self-healing surfaces. Materials designed to change their shape in a controllable and reversible manner have been considered in the production of drug delivery systems,^1^ gene delivery vehicles,^2^ precision actuators and switches,^3−6^ artificial muscles,^7^ and walking devices.^8^ Such adaptive soft matter can be classified depending on the triggering mechanism that initiates the shape-changing behavior; this can include temperature,^9^ electric charge,^3,7,10^ humidity,^6,11−14^ pH,^2^ solvents,^13−16^ or UV light.^17^ The development of such new materials is often inspired by the strategies of living organisms to sense the environment or achieve actuation. As an example, plant systems such as pine cones are able to generate movement by a differential swelling of different parts of their tissue.^5^ Similar mechanisms have been employed in hydrogels to achieve controlled and reversible changes in shape under conditions of changing humidity; however, their response time is usually slow (up to several hours) and the generated stress is low due to high water content.^18^

A variety of bilayer actuators have been fabricated that incorporate active layers on a passive substrate. For example, Ma et al. developed a multilayer film actuator capable of carrying significant loads (0.375 mN; 128 times the actuator’s weight), which was triggered by changes in humidity.^8^ Their actuator exploited the different swelling rates of film layers, thereby generating internal strains. A single-material actuator based on gradient dewetting of water and other solvents was presented by Wu et al.,^16^ where a fast curling response was achieved based on gradient swelling of the material. Zhao et al. developed a hygroscopic actuator capable of bending in response to acetone vapor and humidity by introducing a gradient of porosity through the membrane thickness.^14^ The authors demonstrated that the graded porous structure can yield high forces (0.75 mN; 25 times actuator’s weight) as a result of a gradient desorption mechanism. A limitation of humidity powered devices is their susceptibility to external conditions, thereby requiring a controlled environment. The difficulty in achieving precise control, due to humidity fluctuations, and a slow response time remain significant challenges in the successful implementation of humidity-driven actuators. In addition, many existing polymer actuators are susceptible to humidity and extreme temperatures and require complex manufacturing methodology.

Soft actuators with high contractile force provide a significant advantage in manufacturing soft robots for diverse engineering use. The high contractile force provided by the solvent-driven materials enables the roboticists to design robots with larger deformation and more flexible segment bodies, which provides the opportunities for new robot design without impairing the static and dynamic responses of the robots.

As a result, to develop an efficient and universal soft actuator, there is a need to create actuator materials that are easily manufactured, are capable of rapid and reversible deformation, can deliver high loads, and are able to withstand a wide range of hot/cold temperatures and humidity levels.

Here, we present a composite based on a microporous polymer to create actuators that exhibit rapid, reversible, and repeatable deformation that is accompanied by very high loads in the presence of a solvent (ethanol, acetone, and DMSO), but remain insensitive to water and humidity, and can withstand a large temperature range. The actuator is formed from a polymer of intrinsic microporosity, PIM-1, which exhibits strong shrinkage in the presence of a solvent. PIM-1 is a processable polymer with a unique combination of properties, which includes a high BET surface area (∼800 m^2^/g) and good solvent processability due to its rigid, but kinked, backbone structure which prevents efficient space packing of the polymer chains. This leads to the creation of nanoscale pores smaller than 2 nm in three dimensions.^19^ While the intrinsic microporosity of these materials has attracted significant interest for applications related to separation membranes^20^ and gas storage,^21−23^ we demonstrate in this paper that these intriguing materials can be successfully used as actuators and drug delivery vehicles. To achieve three-dimensional (3D) actuation, PIM-1 is doped with an activated carbon filler, AX21 (Anderson Development Co., Adrian, MI).^24^ As a result of the asymmetric distribution of filler in the composite, a bilayer structure is formed, where each layer produces a different internal strain in response to a solvent. We investigate the influence of solvent type, of the evaporation rate and of the filler content on the magnitude and kinetics of actuation. We present their applicability after being exposed to a range of temperatures and in a water environment. Finally, we demonstrate the concept of a micro-origami capsule using the proposed composite actuator for drug delivery applications.

## 2 Materials and Methods

### 2.1 Polymer and Composite Sample Preparation

PIM-1 was synthesized using the method described previously.^21^ To summarize, 5,5′,6,6′-tetrahydroxy-3,3,3′,3′-tetramethyl-1,10-spirobisindane (5.11 g, 15 mmol, 1 equiv), tetrafluoroterephthalonitrile (3.00 g, 15 mmol, 1 equiv), and anhydrous K_2_CO_3_ (16.59 g, 120 mmol, 8 equiv) were used as starting materials. The solids were evacuated and backfilled three times with nitrogen and 100 mL of anhydrous dimethylformamide (DMF) was added, after which precipitation was achieved while stirring for 3 days at 65 °C under N_2_. The solution was then hydrated, filtered, air-dried, and purified via reprecipitation. The final step was dissolving PIM-1 in chloroform and reprecipitating three times with MeOH before drying under vacuum at 80 °C overnight.

The number-average molar mass (*M*_n_) of the obtained PIM-1 measured with gel permeation chromatography (GPC) in the previous study was 76 261 g/mol, weight-average molar mass (*M*_w_) was 193,074 g/mol, and dispersity index *Đ* = 2.5.^21^

AX21 was prepared from a mixture of KOH and petroleum coke by activation at 700 °C; the BET surface area is in the range of 2800−3500 m/g.

Polymer films were cast by dissolving PIM-1 (1.22 g) in chloroform (35 mL) and pouring the solution into a large 200 mm Petri dish, where it was left to evaporate for 48 h. To prepare PIM-1/AX21 composite films, the activated carbon AX21 was separately stirred with a small amount of chloroform (5 mL) for 24 h and added to the PIM-1 solution. The mixture was then stirred for another 24 h at room temperature. Different ratios of AX21/PIM-1 mixtures were prepared to obtain 10, 20, 30, 40, and 50 wt % of AX21 in the PIM-1 composites. Solutions were poured into 200 mm Petri dishes and left to evaporate for 48 h. The evaporation rate was optimized to achieve mechanically robust films and to tailor the distribution of filler and actuation capacity. To assess the effect of evaporation rate, composite films with 40 wt % concentration of AX21 filler were evaporated in an open container for 12 h, in a semiclosed container for several days, and in a closed container for a period of 2 weeks. After solvent casting, all films were dried under vacuum at 80 °C for 8 h to eliminate solvent residues. For testing purposes, rectangular samples were cut according to BS EN ISO 527-3:1996 standard (1/2 of specimen type 2).^25^ Preliminary testing was performed to confirm that the decreased dimensions of specimens did not influence the mechanical characterization results. Samples thickness was assessed using a Mitutoyo 227-211 Absolute Digimatic Micrometer (Mitutoyo, Kawasaki, Japan) with ±0.001 mm measurement accuracy and measuring force adjustment.

### 2.2 Imaging of Composite Samples

PIM-1/AX21 composite materials with different concentrations of AX21 in PIM-1 (10, 20, 30, 40, and 50 wt %) were imaged using a scanning electron microscope (SEM) (JSM6480LV, JEOL Ltd., Tokyo, Japan). In addition, samples containing 30 wt % AX21 manufactured with faster and slower evaporation rates (48 h and 2 weeks) were compared to understand the influence of changes in the composite microstructure due to the rate of evaporation and its impact on actuation behavior, in particular, the distribution of the AX21 particles in the composite. The cross sections of the composite samples were obtained by the brittle fracture of composite samples; they were then gold coated, and images of fracture surfaces were acquired.

### 2.3 Contraction and Actuation Force Measurement

To assess the response of pure and dry PIM-1 films to the solvents used for actuation, the dimensions of pure PIM-1 samples were measured, and samples were immersed in ethanol and measured again after evaporation to determine the shrinking magnitude and its repeatability. PIM-1 contractile forces in response to a range of solvents evaporation were measured in tension. Rectangular samples were exposed to three different solvents: ethanol, propan-2-one (acetone), and dimethyl sulfoxide (DMSO, Sigma-Aldrich, St. Louis, MO). Following solvent immersion for 1 min, the samples were clamped at a fixed gauge length of 40 mm in a uniaxial tensile testing machine, an Instron 3369 instrument with a 50 N static load cell (Instron, Norwood, MA). An initial preload of 0.1 N was applied to produce a uniform tension along the sample. Contractile forces produced upon solvent evaporation were measured in the static setup using the load cell with no external strain applied. The force as a function of time was monitored to evaluate the rate of sample shrinkage with time. Three samples were tested with each solvent.

To measure the contractile forces and the influence of AX21 additive concentration on actuation force of the PIM-1/AX21 composites, composites with 10, 20, 30, 40, and 50 wt % of AX21 in PIM-1 were tested in the same tensile testing setup using ethanol as a solvent, and the force as a function of time was recorded. At least five samples were tested for each actuator composition.

### 2.4 Thermal Stability of PIM-1 and PIM-1/AX21 Composites

The ability of the composites to withstand hostile cold and hot conditions was also evaluated. PIM-1 samples and composite samples were subjected to a thermal treatment of 250 °C for 30 min. After heat treatment, they were exposed to ethanol and their swelling and shrinking behavior were monitored. Samples were also exposed to liquid nitrogen for 30 s, then they were left to warm to room temperature, and their actuation behavior was evaluated.

## 3 Results

### 3.1 Shrinkage of Pure PIM-1 and the Autonomous Actuation of PIM-1/AX21 Composites

Pure PIM-1 samples did not exhibit significant actuation prior to solvent treatment; they were entirely transparent, yellow in color, and flat. After solvent wetting, they would simply swell and then shrink upon solvent evaporation, with no out-of-plane bending or curling of the material. The sample length decreased as a result of ethanol evaporation by almost 10% of the original length, 100–91 mm.

The composite samples became increasingly black in color with a higher concentration of activated carbon in the polymer PIM-1 matrix. Samples with 10–20 wt % were yellow and partially transparent with clearly visible activated carbon particles distributed within the yellow polymer matrix. Samples with 30 wt % and higher filler content were almost entirely black and opaque. The bottom surface that was in contact with the glass Petri dish during casting was smooth and matte, while the top surface was rough and glossy in appearance, see Figure 2a. In samples with the higher proportions of AX21, the apparent surface roughness increased. When freshly cast composite films were cut into rectangular strips, they would spontaneously curl into loose helices or half-tubes, depending on the direction of the incision (longitudinal vs transverse), such as in Figure 2a. After solvent exposure, they would return to a flat shape (Figure 2b), and on subsequent evaporation, composites would spontaneously curl into tight tubes around 1–2 mm in diameter (Figure 2c,d). The described behavior was observed in all composites starting from 20 wt % concentration of AX21 filler, up to 50 wt %. Samples containing 10 wt % AX21 in PIM-1 exhibited a lower degree of curling and formed loose helices rather than tubes. These films exhibited autonomous shape changing when exposed to a solvent, which was reversible and repeatable.

**Figure 1:**
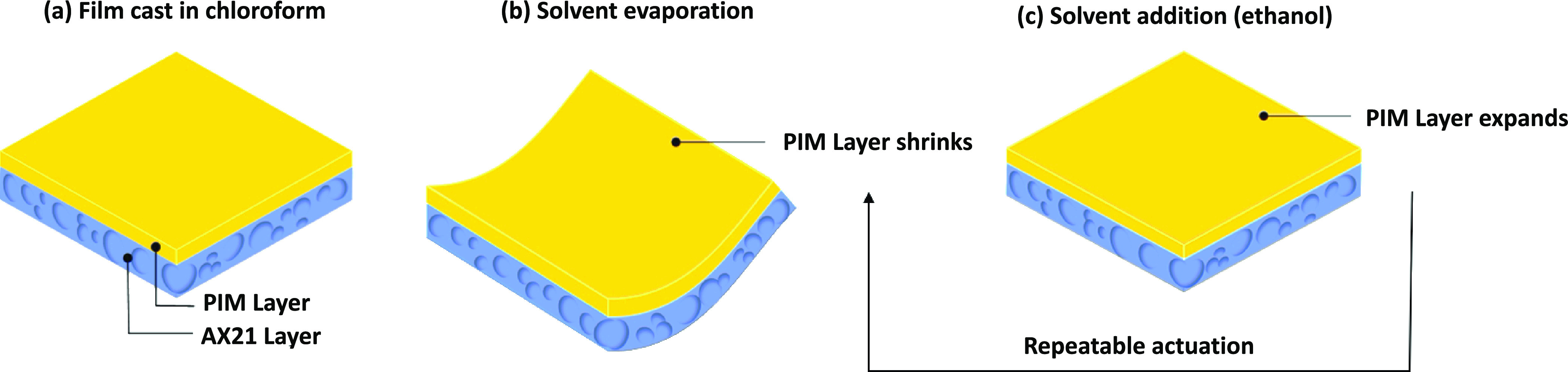
Repeatable actuation mechanism in PIM-1/AX21 composite. (a) Flat samples after casting in chloroform, (b) curling occurring after solvent evaporation, and (c) film flattening upon wetting with the solvent.

**Figure 2:**
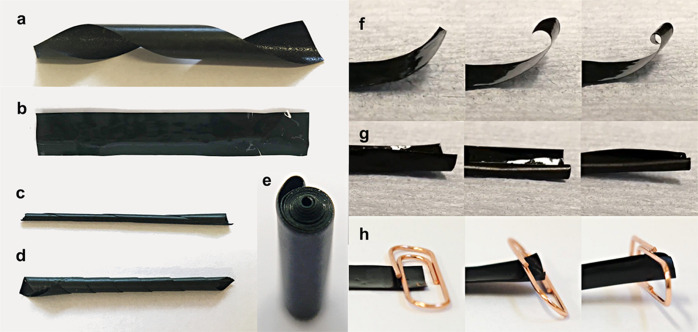
Different modes of autonomous curling and imposed curling in PIM-1/AX21 composites. All samples presented in the figure contained 40 wt % filler. Dimensions of all of the samples in the relaxed state were 10 mm width and 75 mm length. All samples were obtained with 48 h casting evaporation process. The solvent used was ethanol. (a) Sample before ethanol treatment exhibiting helical curling after an oblique incision, (b) wet sample immersed in ethanol, (c) dry sample autonomously curled along its long axis, (d) dry sample autonomously curled along its short axis, (e) dry sample which was forced to curl into a tight tube and maintained the shape after drying, (f) sample autonomously curling along its short axis during ethanol desorption after a transverse incision, (g) sample autonomously curling along its long axis during ethanol desorption after a longitudinal incision, and (h) sample lifting an object over 10 times heavier than itself during autonomous actuation caused by ethanol desorption.

Curling occurred in such a manner that the upper rough surface of a film was always the internal surface of the curled material, and the smooth surface was the outer surface. The curling behavior could be reversed by moistening samples with the solvent, as shown schematically in Figure 1. Upon wetting or soaking in a solvent, samples straightened into flat strips, Figure 2b. Upon solvent evaporation, the samples then exhibited rapid curling into tubes or helices, depending on their initial shape, see Figure 2c,d,f,g. The initial shape, as well as the direction of curling after solvent evaporation, was dependent upon the direction of cutting during sample preparation. Transverse cutting caused the samples to curl along their short axis, see Figure 2f, while longitudinal incision led to curling along its long axis (Figure 2g). An oblique incision produced actuators combining both curling directions leading to the formation of helices, as presented in Figure 2a. This actuation was sufficiently strong to lift objects at least 10 times heavier than the sample itself during the process, Figure 2h. The kinetics of sorption and desorption that led to actuation depended on the nature of the solvent and was faster with acetone than with ethanol. Curled composites placed in acetone straightened into flat strips in less than 1 s, whereas with ethanol, the process took up to 5 s. Upon desorption of acetone, the composite samples began curling after being placed in air for approximately 10 s and required another 15–20 s to produce tightly closed tubes. Samples removed from ethanol started curling after 30 s and needed another 50–100 s for full tube formation, see Supporting Information Video 1. This behavior was repeatable, where the shape of curling was identical during every cycle and could be performed at least 50 times. Films exhibited mechanical robustness in both wet and dry states and were capable of continuous autonomous locomotion by alternating sorption and desorption of a solvent.

### 3.2 Imaging of PIM-1/AX21 Composite Samples

**Figure 3:**
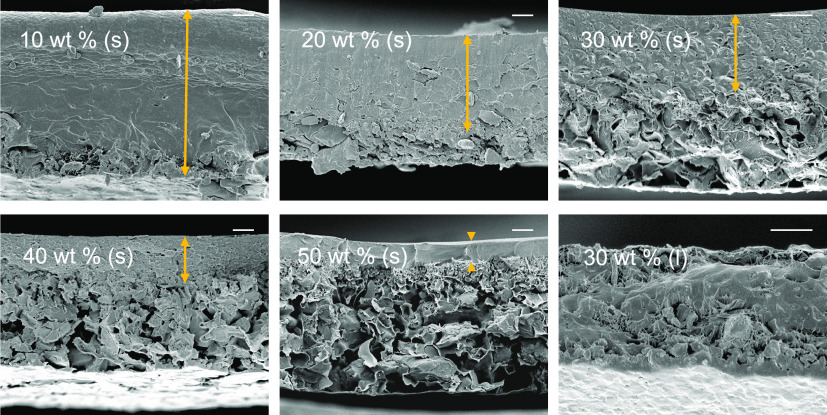
SEM images of the cross sections of PIM-1/AX21 composite samples cast and evaporated over 48 h (s) with different filler concentrations and a 30 wt % sample cast and evaporated over 2 weeks (l); the scale bar in all images is 10 μm in length. Yellow arrows point out the regions with topographies typical of pure PIM-1.

Microstructural analysis of composite samples with different filler concentrations and samples evaporated for 48 h or 2 weeks was performed by SEM, see Figure 3. Samples cast and evaporated over 48 h (Figure 3s) exhibited a distinct bilayer structure with a clear interface between the lower porous and upper dense regions. This structure would form as the activated carbon filler would settle to the bottom of a film during evaporation, and a dense matrix that was characteristic of PIM-1 would be visible at the top of the film. The ratio of the porous filler-rich and filler-poor regions was dependent on the filler content in the composite, and the porous layer thickness would increase with an increase in the concentration of AX21 filler, as indicated by the orange arrows in Figure 3. The distinction between two layers was less apparent in samples that were cast and evaporated over a longer period of 2 weeks (Figure 3l), and their distribution was much more uniform and disorderly. This difference in the structure had an impact on actuation/curling response and will be discussed later in relation to the actuation mechanism.

### 3.3 Measurement of Shrinkage and Contractile Forces

**Figure 4:**
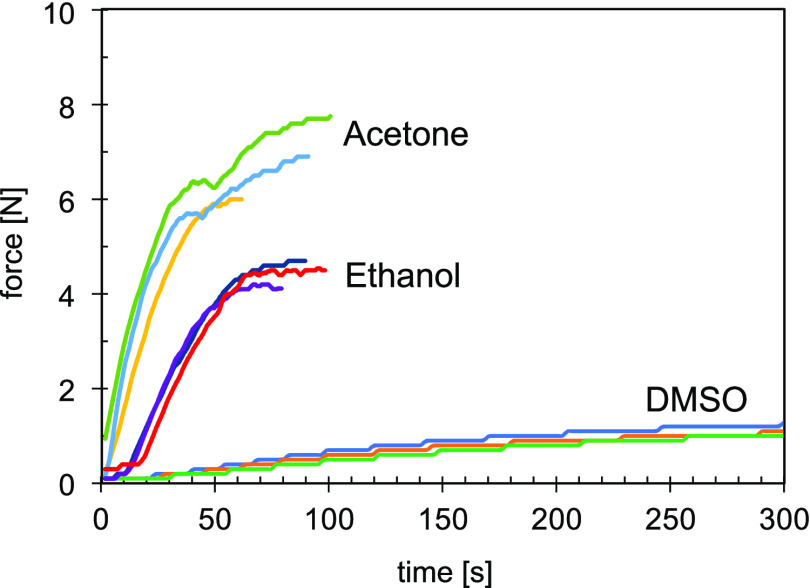
Contractile force of pure PIM-1 upon exposure and evaporation of different solvents.

**Figure 5:**
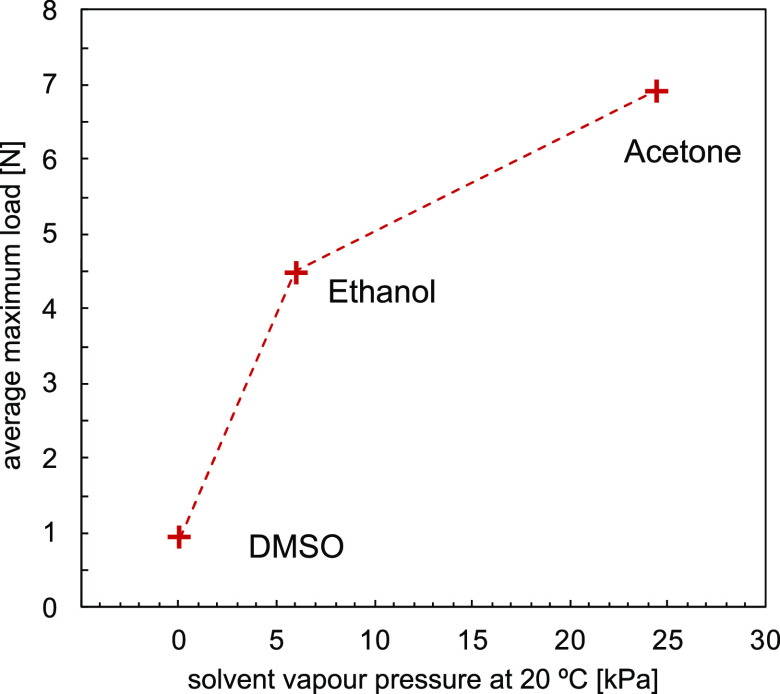
Average maximum load obtained with different solvents vs solvent vapor pressure at 20 °C.

The kinetics and magnitude of shrinking were dependent upon the type of solvent used and were assessed as a contractile force measurement of PIM-1 exposed to ethanol, acetone, and DMSO, as shown in Figure 4. The maximum force was obtained for samples that had been immersed in acetone (7.8 N). The average force for samples exposed to acetone was almost 7 N, whereas for ethanol, it was 4.5 N. DMSO had the lowest influence on the contractile force as samples produced only 1 N upon DMSO evaporation. When the magnitude of maximum average load obtained with each type of solvent is plotted against solvent vapor pressure at 20 °C, as in Figure 5, it can be seen that the measured maximum force increased with an increase of vapor pressure. Acetone and ethanol both required 100 s to achieve maximum force, which was higher for acetone. Using DMSO, the maximum value of force was achieved after over 400 s.

**Figure 6:**
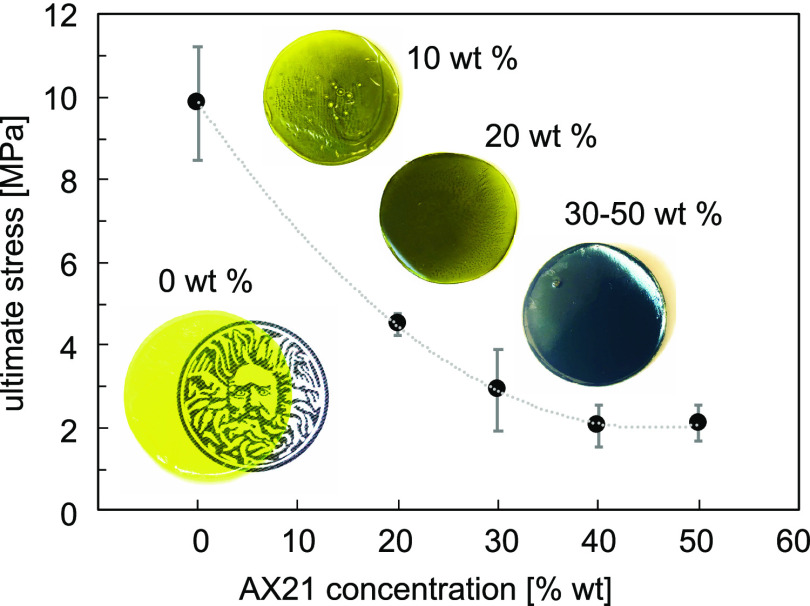
Average ultimate stress obtained from spontaneous contractile force measured for different concentrations of AX21 in PIM-1 with error bars representing standard deviations. The curve is provided to guide the eye only. Insets: (bottom left corner) pure PIM-1 is perfectly transparent; (right top corner from top to bottom): representative images of PIM-1/AX21 samples with different concentrations: 10, 20, and 30–50 wt % of AX21 in PIM-1. Samples with 30 wt % and more AX21 were uniformly black.

Contractile forces measured in pure PIM-1 and PIM-1/AX21 composites upon solvent desorption in some cases were sufficient to cause mechanical failure of the materials if they were constrained in the mechanical test machine. Pure PIM-1 samples produced a stress of up to 12 MPa, associated with solvent evaporation. The highest ultimate force and stress for composite samples exposed to ethanol were obtained for specimens containing 10 wt % AX21 (3.18 N and 5.42 MPa) and 20 wt % AX21 (3.21 N and 4.93 N). The ultimate tensile strength of the composites decreased with the increasing concentration of AX21 (Figure 6). However, the average stress values obtained for both 40 and 50 wt % samples were similar (2.04 and 2.11 MPa, respectively). The pure PIM-1 sample failed at a maximum force 5.43 N in ethanol and up to 7.8 N in acetone, which is an equivalent of over 20 000 times the weight of the sample (0.035 g).

### 3.4 Thermal Stability of PIM-1 and PIM-1/AX21 Composites

PIM-1 samples removed from the oven at temperature 250 °C remained mechanically robust and exhibited the same swelling and shrinking behavior. Composite samples did not exhibit any changes in actuating behavior after the same thermal treatment (see Supporting Information Video 2). After treatment with liquid nitrogen, no degradation in actuation behavior was observed (see Supporting Information Video 3) and samples subjected to both heating and liquid nitrogen treatment also maintained their actuation capabilities (see Supporting Information Video 4).

## 4 Discussion

PIM-1 exhibits swelling and shrinking behavior upon exposure to solvents (ethanol, acetone, and DMSO) but remains insensitive to water. Shrinking after ethanol exposure led to a 12% decrease in sample length and forces with a magnitude over 20,000 times the weight of the sample, which is higher than previously reported.^6,8,11,12,14^ While pure PIM-1 does not exhibit out-of-plane bending, PIM-1/AX21 composites exhibit tubular curling behavior and have the capacity to be formed into desired shapes (Figure 2e), where the behavior is reversible and repeatable. The demonstrated phenomenon can be of use for a number of applications in the fields of actuation, sensing, soft robotics, and artificial muscles. Three-dimensional actuation was specific for PIM-1/AX21 composites and was not observed in pure PIM-1 films or composites with another filler such as fine particles of porous aromatic framework PAF-1.^22^

A similar mechanism of swelling-induced autonomous motility was previously observed in response to humidity.^6,12,14^ The curling was dependent on the direction of sample incision as previously observed by Douezan et al.^26^ However, many actuators that exhibit solvent-induced actuation are also triggered by moisture and humidity^14−16^ and are therefore susceptible to changes in environmental conditions and are applicable only in controlled conditions. Zhao and co-workers developed a concept for a humidity sensitive membrane consisting of cationic poly(ionic liquid) polymer with a gradient of porosity that bends in the presence of acetone vapor.^14^ The membrane bends into a loop when placed in acetone vapor and quickly recovers its initial flat shape when back in air. The process we demonstrated here is the opposite—the curled shape is the default state of the dry actuator (Figure 2c,d) and the relaxation of an actuator occurs upon exposure to an organic solvent (Figure 2b). Such a process is particularly attractive for utilizing in delivery applications where a substance is enclosed inside a polymer tube and needs to be released upon solvent exposure. Additionally, the speed of the release can be controlled by adjusting the type of a solvent as the actuation kinetics vary for DMSO, acetone, and ethanol. The difference in curling speed can be attributed to varying boiling points (189, 56, and 78 °C, respectively) and volatility of the used solvents. A higher vapor pressure solvent evaporates faster, which leads to a rapid increase in the contractile force that causes more dynamic actuation. The magnitude of actuation has shown to be solvent vapor pressure dependent, thus could be controlled by adjusting solvent parameters. Therefore, the proposed solvent-driven actuator is programmable which enables the controllable process of drug delivery as one of potential application areas with release upon solvent local application.

Multiple aspects of the curling behavior of PIM-1/AX21 actuators are highly controllable and can be programmed. The direction of curling can be controlled by the direction of an incision during the sample preparation process. Transverse cutting leads to curling along its short axis, longitudinal cutting produces samples curling along their long axis into tubes, and oblique incision induces combined curling behavior and the formation of helices. The magnitude of curling depends on the evaporation duration during the solvent casting procedure. A slower evaporation of 2 weeks led to lower magnitudes of curling, as compared to higher magnitude after fast evaporation (48 h). The contractile forces produced by the actuators are dependent on the amount of AX21 filler inside the PIM-1 matrix and the vapor pressure characteristics of a triggering solvent used. The solvent type also influences the speed of actuation.

### 4.1 Actuation Mechanism

Our actuator materials use microporous polymer composites to obtain robust actuators with large actuation capacity. The curling behavior of the composites can be explained by the presence of a gradient of carbon particles through the sample thickness, see Figure 3; it is noted that curling did not occur in pure PIM-1 or in our previous work on PIM-1 composites with homogeneous distribution of a filler.^22^ A porosity gradient has been shown to lead to bending behavior in polymer composite membranes due to an asymmetry of strain.^14^ The gradients in our PIM-1/AX21 composites are formed due to range of particle sizes of the activated carbon filler and during the solvent casting-evaporation process; the larger carbon particles fall to the bottom of the chloroform solution, thus creating a bilaminar film structure in the case of rapid evaporation after casting, see Figure 3. We propose that the lower carbon-rich layer swells and shrinks to a lesser extent than the upper PIM-1-rich layer (Figure 1), and this is in agreement with the observation that the lower smooth layer would always form the outer surface of the tube and the upper layer would form the inner layer of the tube. It is also in agreement with the pure PIM-1 creating the largest forces on shrinkage when constrained in a tensile test machine, Figure 6. Finally, composites formed by slow evaporation after casting have exhibited a more even distribution of carbon, see Figure 2f, and produce a smaller degree of curling; the more homogeneous distribution of filler may be a result of rearrangement of polymer chains and carbon particles in the solution due to slowly increasing density. The evaporation rate during casting can therefore be employed to control the curling magnitude. Additionally, the curvature of bending had been described to scale inversely with membrane thickness,^14^ which can be considered another parameter enabling full control over an actuator’s curling behavior.

PIM-1 has shown no sensitivity to water due to its strong hydrophobic nature, which explains why it interacts differently with organic solvents than with water. As PIM-1 does not exhibit swelling in water, it is clear that organic solvents have stronger interactions with the polymer when compared to water, which results in enhanced swelling behavior when using ethanol, acetone, or DMSO.

Another mechanism of interest was reported by Ma et al. who found that a pentaerythritol ethoxylate-polypyrrole (PEE-PPy) composite increased its elastic modulus between moist and dry states, which they explained by the polymer structure being weakened upon water sorption, which recovered upon desorption.^6^ This mechanism could explain the decreasing brittleness of PIM-1 films and composites when soaked in ethanol.

### 4.2 Comparison and the Unique Properties of PIM-1/AX21 Actuators

Zhao and co-workers measured a maximum force of 0.75 mN produced by their actuator, which was 25 times the weight of the 3 mg actuator.^14^ Zhang described a moisture-responsive graphene oxide-based actuator that could lift objects 50 times heavier.^15^ The water gradient-driven composite actuator presented by Ma et al. has shown an impressive capacity of lifting objects 380 times the weight of the actuator.^6^ In this work, the maximum load achieved during contractile force measurement of pure PIM-1 was 7.8 N, which is an equivalent of over 20 000 times the weight of a sample (35 mg). The maximum load achieved for a PIM-1/AX21 composite actuator reached 3.18 N constituting 9000 times the weight of the sample. Both values are much higher than previously reported in the literature for humidity and solvent responsive actuators.^6,8,11,12,14^ Actuators with such strength can be used as soft robotics joints in the development of walking devices, and the continuous swelling and contraction of the polymer could be employed to drive a piezoelectric element to power low-power devices via energy harvesting. High contractile force solvent-driven actuator in drug delivery applications would be able to carry more substance to the target locations.

The demonstrated actuator development methodology is applicable to composites with other types of additives that could introduce additional functionalities, whereas the simple solution processability enables employing other fabrication technologies such as electrospinning or 3D printing of structures with complex combined functionalities.

It has been shown that porosity can increase the accessibility of solvents and promote mass transfer.^27^ We propose that the high contractile forces achieved by the presented actuators are possible due to the microporous characteristics of the components and the combined high surface area of the composite, which facilitates the access and efficient mass transfer of the organic solvents within the structure and can enhance the kinematics and magnitude of actuation. Further analysis of how porosity, pore size, and shape influence the kinematics of the solvent-driven actuator can provide insights into optimization of the actuator and further possible applications.

What distinguishes our proposed actuator from other existing concepts^6,8,11,12,14^ is its stability in water, a feature necessary for artificial muscles or drug delivery vehicles to be applied in wet or humid environments. As an example, Zhao et al. report that their actuators can be triggered by a combination of solvent vapor and humidity,^14^ whereas the PIM-1/AX21 actuator responds only to organic solvents (ethanol, acetone, and DMSO) while remaining inactive in water. As it cannot be triggered by humidity or the addition of water, it can be placed in a humid environment or in water while still being able to be triggered on demand (by solvent addition) as opposed to uncontrollable environmental changes. One of the proposed applications for this type of material is in sensing the presence of a solvent and its concentration in a water solution. In addition, we have previously shown that PIM-1 alone has excellent thermal stability, does not become brittle in liquid nitrogen, and is stable between −150 and 350 °C;^21^ the PIM-1/AX21 composites start decomposing at ∼350 °C.^23^ In this study, we have demonstrated resistance to the thermal treatment of PIM-1 and PIM-1/AX21 composites and that their actuating behavior remains unchanged after heat treatment at 250 °C for 30 min and treatment with liquid nitrogen (−196 °C), which provides a wide range of operational temperatures. This excellent thermal stability makes the actuators suitable for applications in high-temperature environment or for high-temperature treatment such as steam sterilization necessary for medical applications. Their mechanical stability in low temperatures allows for the long-term storage of micro-origami capsules loaded with drugs prior to application.

## 5 Micro-Origami Capsule Demonstration

**Figure 7:**
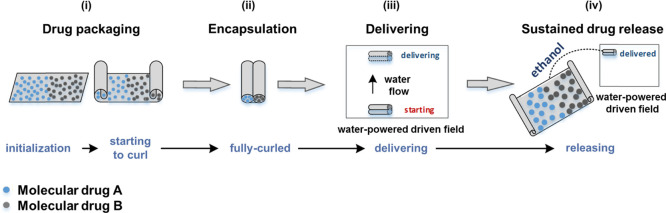
Schematic of micro-origami capsule for drug encapsulation and delivery. The capsule can carry two different types of drugs at the same time because of the advantages of the composite actuator, which can naturally form two curled ends. The water-powered driven field is used for capsule delivery due to its insensitivity to humidity and water.

**Figure 8:**
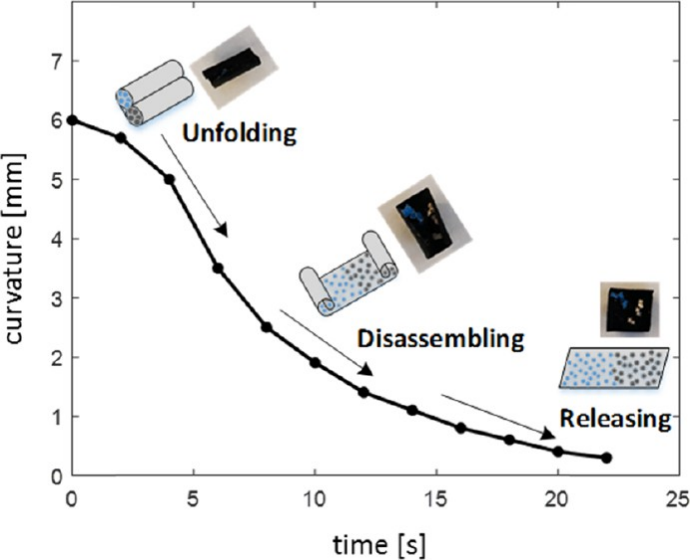
Curvature of the capsule vs ethanol desorption time.

We now develop a demonstration for a proposed application of the actuator material as a drug delivery micro-origami capsule (see Figure 7 and Supporting Information Video 5), where the external diameter of the capsule was 1.5 mm and the capsule length 10 mm. A 40 wt % PIM-1/AX21 composite sample was used in the demonstration due to its high degree of actuation. Two different particle types (to represent potential drugs A and B) were loaded into the uncurled composite actuator after it was immersed in ethanol at room temperature (Figure 7i) and then encapsulated autonomously along with the desorption of ethanol, Figure 7ii. The capsule can carry two different types of drugs that need to be separated from each other. After the completion of encapsulation, the actuator was delivered via water-powered driven flow to the target site, Figure 7iii. When the capsule reached the targeted site, ethanol was applied to open the micro-origami capsule for release, Figure 7iv. The corresponding folding curvature of the capsule vs ethanol desorption time is shown in Figure 8, where the whole process excluding delivery takes 22 s. The capsule is fully curled from both ends naturally with two separated enclosed chambers (external diameter = 1.5 mm). The larger or smaller capsule can be fabricated for various drug delivery applications.

**Figure 9:**
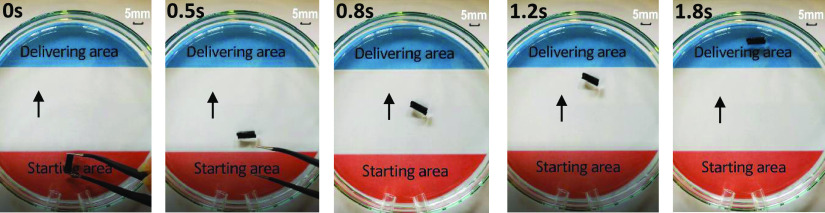
Capsule delivery using a water-powered driven field.

The capsule is water-resistant, and thus, the water-powered flow can effectively deliver the capsule to the target site, as shown in Figure 9. The delivery speed is determined by the power flow, which can be adjusted for various requirements.

## 6 Conclusions

We have presented a class of composite polymer-based actuators and an approach to produce actuators that can be controlled by the presence of solvents. The magnitude and speed of actuation can be controlled by adjusting the amount of activated carbon filler added to the polymer matrix, the rate of solvent evaporation, and the nature of the solvent. We have shown that the highest reported contractile forces observed in polymer actuators triggered by a solvent other than water. Due to its insensitivity to water and humidity and its stability to high and low temperatures, the actuator can be applied in a range of environments and could be used as a solvent sensor. The inverse contractile mechanism in dry air makes the material of interest as a controlled delivery and release vehicle. We have demonstrated that the proposed actuator can be used as a micro-origami capsule for drug delivery, where the actuator could enclose two types of drugs, deliver them in a water environment, and release them on demand in a desired location. The demonstration showed potential for significant advantages, such as eliminating drug leakage compared to current technologies.

## Supporting Information Available

PIM-1/AX21 composite actuation. The video shows curling of PIM-1/AX21 sample with 40 wt % of AX21 in PIM-1 into a tight tube during ethanol desorption. Speed increased 8-fold (Video S1) (MP4)
Actuator behavior after heat treatment at 250 °C for 30 min remains unchanged. Presented on the example of application as a drug delivery vehicle. Speed increased 32-fold (Video S2) (MP4)
Actuator behavior after immersing in liquid nitrogen remains unchanged. Presented on the example of application as a drug delivery vehicle. Speed increased 32-fold (Video S3) (MP4)
Actuator behavior after heat treatment and liquid nitrogen treatment remains unchanged. Presented on the example of application as a drug delivery vehicle. Speed increased 32-fold (Video S4) (MP4)
Drug delivery demonstration. Actuator is loaded with two kinds of drugs; it encapsulates the drugs in two separate chambers during ethanol desorption. Delivery of the capsule to the application site is achieved via water-powered driven field. After delivery, the micro-origami capsule is opened with ethanol and the drugs are released (Video S5) (MP4)

## References

[ref1] Caldorera-Moore M. E.; Liechty W. B.; Peppas N. A. Responsive Theranostic Systems: Integration of Diagnostic Imaging Agents and Responsive Controlled Release Drug Delivery Carriers. Acc. Chem. Res. 2011, 44, 1061–1070. 10.1021/ar2001777. 21932809 PMC3219056

[ref2] Benns J. M.; Choi J. S.; Mahato R. I.; Park J. S.; Kim S. W. PH-Sensitive Cationic Polymer Gene Delivery Vehicle: N-Ac-Poly(L-Histidine)-Graft-Poly(L-Lysine) Comb Shaped Polymer. Bioconjugate Chem. 2000, 11, 637–645. 10.1021/bc0000177. 10995206

[ref3] Smela E. Conjugated Polymer Actuators for Biomedical Applications. Adv. Mater. 2003, 15, 481–494. 10.1002/adma.200390113.

[ref4] Smela E. Conjugated Polymer Actuators. MRS Bull. 2008, 33, 197–204. 10.1557/mrs2008.45.

[ref5] Fratzl P.; Barth F. G. Biomaterial Systems for Mechanosensing and Actuation. Nature 2009, 462, 442–448. 10.1038/nature08603. 19940914

[ref6] Ma M.; Guo L.; Anderson D. G.; Langer R. Bio-Inspired Polymer Composite Actuator and Generator Driven by Water Gradients. Science 2013, 339, 186–189. 10.1126/science.1230262. 23307738 PMC3635810

[ref7] Must I.; Kaasik F.; Põldsalu I.; Mihkels L.; Johanson U.; Punning A.; Aabloo A. Ionic and Capacitive Artificial Muscle for Biomimetic Soft Robotics. Adv. Eng. Mater. 2015, 17, 84–94. 10.1002/adem.201400246.

[ref8] Ma Y.; Zhang Y.; Wu B.; Sun W.; Li Z.; Sun J. Polyelectrolyte Multilayer Films for Building Energetic Walking Devices. Angew. Chem. Int. Ed. 2011, 50, 6254–6257. 10.1002/anie.201101054. 21598368

[ref9] Hu Z.; Zhang X.; Li Y. Synthesis and Application of Modulated Polymer Gels. Science 1995, 269, 525–527. 10.1126/science.269.5223.525. 17842364

[ref10] Sun Z.; Yang L.; Zhang D.; Song W. High Performance, Flexible and Renewable Nano-Biocomposite Artificial Muscle Based on Mesoporous Cellulose/Ionic Liquid Electrolyte Membrane. Sens. Actuators, B 2019, 283, 579–589. 10.1016/j.snb.2018.12.073.

[ref11] Zhang Y.; Jiang H.; Li F.; Xia Y.; Lei Y.; Jin X.; Zhang G.; Li H. Graphene Oxide Based Moisture-Responsive Biomimetic Film Actuators with Nacre-like Layered Structures. J. Mater. Chem. A 2017, 5, 14604–14610. 10.1039/c7ta04208f.

[ref12] Zhang L.; Liang H.; Jacob J.; Naumov P. Photogated Humidity-Driven Motility. Nat. Commun. 2015, 6, 7429 10.1038/ncomms8429. 26067649 PMC4490392

[ref13] Zhang K.; Geissler A.; Standhardt M.; Mehlhase S.; Gallei M.; Chen L.; Thiele C. M. Moisture-Responsive Films of Cellulose Stearoyl Esters Showing Reversible Shape Transitions. Sci. Rep. 2015, 5, 11011 10.1038/srep11011. 26051984 PMC4458881

[ref14] Zhao Q.; Dunlop J. W. C.; Qiu X.; Huang F.; Zhang Z.; Heyda J.; Dzubiella J.; Antonietti M.; Yuan J. An Instant Multi-Responsive Porous Polymer Actuator Driven by Solvent Molecule Sorption. Nat. Commun. 2014, 5, 4293 10.1038/ncomms5293. 24980606

[ref15] Liu J. C.; Shang Y. Y.; Zhang D. J.; Xie Z.; Hu R. X.; Wang J. X. Single-Material Solvent-Sensitive Fluorescent Actuator from Carbon Dots Inverse Opals Based on Gradient Dewetting. Chin. J. Polym. Sci. 2017, 35, 1043–1050. 10.1007/s10118-017-1981-y.

[ref16] Wu H.; Kuang M.; Cui L.; Tian D.; Wang M.; Luan G.; Wang J.; Jiang L. Single-Material Solvent-Sensitive Actuator from Poly(Ionic Liquid) Inverse Opals Based on Gradient Dewetting. Chem. Commun. 2016, 52, 5924–5927. 10.1039/c6cc01442a. 27055537

[ref17] Qin C.; Feng Y.; Luo W.; Cao C.; Hu W.; Feng W. A Supramolecular Assembly of Cross-Linked Azobenzene/Polymers for a High-Performance Light-Driven Actuator. J. Mater. Chem. A 2015, 3, 16453–16460. 10.1039/C5TA01543J.

[ref18] Ionov L. Hydrogel-Based Actuators: Possibilities and Limitations. Mater. Today 2014, 17, 494–503. 10.1016/j.mattod.2014.07.002.

[ref19] Budd P. M.; Ghanem B. S.; Makhseed S.; McKeown N. B.; Msayib K. J.; Tattershall C. E. Polymers of Intrinsic Microporosity (PIMs): Robust, Solution-Processable, Organic Nanoporous Materials. Chem. Commun. 2004, 4, 230–231. 10.1039/b311764b. 14737563

[ref20] McKeown N. B. Polymers of Intrinsic Microporosity. ISRN Mater. Sci. 2012, 2012, 1–16. 10.5402/2012/513986.

[ref21] Polak-Kraśna K.; Dawson R.; Holyfield L. T.; Bowen C. R.; Burrows A. D.; Mays T. J. Mechanical Characterisation of Polymer of Intrinsic Microporosity PIM-1 for Hydrogen Storage Applications. J. Mater. Sci. 2017, 52, 3862–3875. 10.1007/s10853-016-0647-4. 32355363 PMC7175681

[ref22] Rochat S.; Polak-Kraśna K.; Tian M.; Holyfield L. T.; Mays T. J.; Bowen C. R.; Burrows A. D. Hydrogen Storage in Polymer-Based Processable Microporous Composites. J. Mater. Chem. A 2017, 5, 18752–18761. 10.1039/c7ta05232d.

[ref23] Tian M.; Rochat S.; Polak-Kraśna K.; Holyfield L. T.; Burrows A. D.; Bowen C. R.; Mays T. J. Nanoporous Polymer-Based Composites for Enhanced Hydrogen Storage. Adsorption 2019, 25, 889–901. 10.1007/s10450-019-00065-x.

[ref24] Rouquerol J.; Rouquerol F.; Llewellyn P.; Maurin G.; Sing K. S. W. Adsorption by Powders and Porous Solids: Principles, Methodology and Applications, 2nd ed.; Elsevier: Amsterdam, 2013.

[ref25] ISO. ISO 527-3: 2018 Plastics—Determination of Tensile Properties—Test Conditions for Films and Sheets, 2018.

[ref26] Douezan S.; Wyart M.; Brochard-Wyart F.; Cuvelier D. Curling Instability Induced by Swelling. Soft Matter 2011, 7, 1506–1511. 10.1039/c0sm00189a.

[ref27] Lee J.-S. M.; Cooper A. I. Advances in Conjugated Microporous Polymers. Chem. Rev. 2020, 120, 2171–2214. 10.1021/acs.chemrev.9b00399. 31990527 PMC7145355

